# Addressing gaps and enhancing experiences in support services for families of pediatric cancer survivors

**DOI:** 10.1038/s41390-024-03320-2

**Published:** 2024-06-17

**Authors:** Verena Paul, Laura Inhestern, Désirée Sigmund, Jana Winzig, Stefan Rutkowski, Gabriele Escherich, Corinna Bergelt

**Affiliations:** 1 Department of Medical Psychology, University Medical Center Hamburg-Eppendorf, Martinistraße 52, 20246 Hamburg, Germany; 2 Department of Pediatric Hematology and Oncology, University Medical Center Hamburg-Eppendorf, Martinistraße 52, 20246 Hamburg, Germany

## Abstract

**Background:**

Childhood cancer’s enduring challenges extend beyond primary therapy. Diverse medical and psychosocial services are available to assist families in follow-up care. This interview study sought to gain a thorough understanding of family motives, satisfaction levels, and barriers to utilization.

**Methods:**

The design of this cross-sectional study involves a qualitative content analysis of semi-structured interviews. We interviewed parents of minor cancer survivors within the first 5 years after primary treatment.

**Results:**

Survivors readily accessed medical support services when necessary. While parents expressed overall satisfaction with the available services, there was a notable gap in their knowledge regarding appropriate psychosocial and family-orientated services. Barriers to access included geographical distances, time constraints, and the absence of childcare options.

**Conclusion:**

There are familial challenges and burdens that fall outside the scope of conventional care services. Tailoring services to family-centered needs, providing more information and easier access to interventions might help to reduce barriers.

**Impact:**

- Existing need notwithstanding, families did not frequently utilize psychosocial services as they did medical ones.
- Identified barriers included lack of awareness, limited availability, long distances, and scheduling conflicts.
- While many studies primarily focus on adult patients or young adults, the present study examines the gaps and strengths in follow-up care for pediatric cancer survivors and their families.
- By acknowledging and addressing the unique challenges and strengths of families with pediatric cancer survivors, we can lead to a more tailored and effective follow-up approach that can enhance their overall well-being by minimizing barriers and providing targeted support.

## Introduction

Surviving childhood cancer is associated with both physical and emotional challenges for the entire family.^1^ Due to aggressive treatment, long hospitalization, and isolation periods, patients are at increased risk of experiencing late effects which can include infertility, cognitive deficits, developmental delays, and endocrine disorders.^2,3^ Those impact their ability to participate in activities of daily living, school, and sports,^4,5^ Further, childhood cancer may cause chronic distress, worse health-related quality of life, and reduced well-being.^6^ A review of the literature implicates that survivors may have difficulties in socializing and forming peer relationships due to insufficient social and emotional skills.^7^ Psychosocial consequences during follow-up care additionally affect the immediate family. Siblings are confronted with emotional distress and adjustment to changed family dynamics.^8^ Parents experience a high level of emotional distress, fatigue as well as an increased vulnerability to depression and post-traumatic stress disorder.^9^

Although taking part in everyday life implies normalcy, it requires a significant amount of energy and resource.^10^ The process of reintegration is associated with further challenges, for instance, changes in working life or (re)integration into kindergarten and school.^11^ Parental emotional stability and family cohesion are crucial for the positive development and successful reintegration of survivors and siblings into everyday life.^12^

The *International Society of Pediatric Oncology (SIOP)* recommends that psychosocial issues should be addressed during and after treatment.^13^ In Germany, according to the decision of the Federal Joint Committee, psychosocial support is to be provided to patients during therapy.^14^ However, there are no mandatory offerings for family members, and there is also no nationwide, insurance-funded support available in follow-up care. Follow-up care begins immediately after primary treatment, initially following a 5-year protocol focused on early detection of relapses and specific late effects such as ototoxicity, cardiotoxic effects, and kidney damage.^15^ After 5 years, the standardized care ends, and further follow-up care is left to the discretion of the treating clinic and the parents and patients, without established guidelines.

However, there is currently no centralized body that systematically integrates medical follow-up care with therapeutic and psychosocial follow-up services. Hence, families, who are often highly burdened by the disease and its consequences, face the challenge of identifying various medical, therapeutic, and psychosocial services, finding contacts, and support options for functional, psychological, social, or school-related. It remains unclear to what extent families are able to meet these challenges and which support options are known and actually utilized. While the issue of adequate utilization (known as “met needs” and “unmet needs”) and factors influencing utilization in adult cancer survivors has been a central research question for several years,^16–18^ there is limited knowledge for children with the disease and their families after completing primary medical treatment. Nevertheless, this information is essential for needs-based care planning, allocation, and utilization with limited resources.

The effectiveness of medical and psychosocial interventions has been demonstrated in several studies.^1^ However, most of the previous interventions were limited to pilot projects that are rarely integrated into the daily follow-up care. Family-oriented rehabilitation (FOR), a specialized approach in Germany since 1985, currently represents the sole psychosocial intervention that has been widely integrated into standardized care. It involves integrating parents and siblings into the inpatient rehabilitation process to maximize the patient’s recovery.^19^ Hence, there is a risk, that important needs remain unmet.^18^ Further reasons for unmet needs can be derived from studies of adolescents and young adults with cancer that indicated a lack of knowledge, a lack of regional and need-based offers, long distances, and a high time requirement as barriers for utilization.^20^

The present study aims to provide an overview of the care situation and the use of medical and psychosocial services from the perspective of caregivers of children with cancer within the first 5 years of follow-up. We investigated the following research questions:

1. In which domains of life do families require support services after primary therapy?
2. What medical and psychosocial follow-up services do families of childhood cancer survivors utilize?
3. To what extent are families of childhood cancer survivors satisfied with the medical and psychosocial follow-up services they receive?
4. What are barriers preventing families from accessing follow-up services?

## Methods and analysis

This interview study was part of the project *“Families with children with cancer after completion of intensive therapy: analysis of biopsychosocial needs and implications for care”* (trial registration number: DRKS00025289). It is funded by the Innovations Fund of the Federal Joint Committee and conducted within the endowed professorship for health care research in pediatric rare diseases (Kindness for Kids professorship).The study protocol was published elsewhere.^21^ The study was approved by a Local Psychological Ethics Committee (LPEK-0281).

### Design

We conducted semi-structured interviews using an interview guideline on the following topics: Utilization of care services, challenges of all family members in everyday life, barriers to utilization, and experiences after completion of primary medical treatment. The manuscript follows the Consolidated Criteria for Reporting Qualitative Research (COREQ).^22^ The checklist can be obtained from the supplements.

### Participants

Interviews were conducted with parents of cancer survivors between 0 and 17 years of age within the first 5 years after the end of primary therapy. All malignant tumor entities were considered. Furthermore, a signed informed consent form and sufficient knowledge of German were required. Participants were recruited either in person through routine medical follow-up consultations or via e-mail through a psychosocial service association. A total of 98 individuals were invited to participate in the interview, of whom 74 signed and returned the consent form of participation. We included a consecutive sample until data saturation was reached. Thus, we obtained a sample size of 30 parents.

### Data collection and analysis

The interviews were conducted via telephone and audio-recorded by VP and DS. Both are female psychologists (M.Sc.), experienced in conducting as well as evaluating qualitative studies, and supervised by LI (Prof, Ph.D.). Participants were informed about the background of the study as well as data protection and organizational matters (e.g., audio recording). Furthermore, sociodemographic data and general information about the survivor’s diagnosis and treatment were collected. Field notes were made during and after the interview. Parents were encouraged throughout to bring their issues to the interview. The interview guide was pilot-tested but only slightly adapted so that the pilot interview could also be included in the analysis. The interviews lasted 57 min on average (Range between 40 and 106 min).

The audio recordings were transcribed verbatim and analyzed using a qualitative structuring content analysis according to Mayring.^23^ Participants were not provided with the transcripts for review or correction. Content categories were first formed inductively from 10 of the interviews and a coding scheme with anchor examples was created. To ensure validity, DS and VP independently applied the coding scheme to an additional set of 10 interviews. Ambiguous coding was discussed and the coding guide was adapted and optimized accordingly. VP performed the final analysis of all material using MAXQDA software. DS coded 15 interviews to enhance reliability. The intercoder agreement between VP and DS was 85%. Sociodemographic and medical characteristics were analyzed descriptively with the software *IBM SPSS Statistics 25*. After the analysis, the interviews were translated into English by VP. The translation was reviewed by DS.

## Findings

### Sample characteristics

30 parents (10 men, 20 women) from 28 families participated. All participants lived in Germany and received medical care through the German healthcare system. Further sample characteristics are displayed in Table 1.

**Table 1:** Sociodemographic data of 30 parents and 28 cancer survivors of 28 families.

Parents	Total (*n* = 30)		Fathers (*n* = 10)		Mothers (*n* = 20)	
	*n*	%	*n*	%	*n*	%
age in years (mean, SD/range)	30	43 (33–55)	10	44 (38–50/4,69)	20	42 (33–55)
familial situation						
number of children (mean, SD/range)	2,1	0,7 (1–4)	2	0.9 (1–4)	2,2	0,5 (1–3)
permanent relationship	28	93	10	100	18	85
living with the other parent and the child/children	25	83	10	100	15	75
living only with the child/children	2	7	0	0	2	20
birth country						
Germany	25	83	9	90	16	80
school education						
>10 years	20	67	5	50	15	55
≤10 years	10	33	5	50	5	45
employment status						
full-time	4	13	4	40	-	-
part-time	21	77	4	40	15	85
not employed	4	7	2	20	2	10
parental leave	1	3	0	0	1	5
cancer survivor	total (*n* = 28)		boys (*n* = 18)		girls (*n* = 10)	
	*m*	sd/range	*m*	sd/range	*m*	sd/range
age (mean, SD/range)	8,6	3,8 (3–17)	9,3	3,8 (4–17)	7	3,5 (3–14)
age at diagnosis (mean, SD/range)	5,8	4,1 (1–15)	6,7	4,2 (1–15)	3,9	3,1 (2–11)
time since diagnosis (years)	3,4	1,9	3,1	1,9	4	1,9
diagnosis	*n*	%	*n*	%	*n*	%
leukemia	10	36	6	33	4	40
CNS^a^-tumor	4	14	3	17	1	10
lymphoma	5	18	5	28	0	0
others^b^	9	32	4	22	5	50
treatment						
chemotherapy	29	97	19	95	9	90
radiotherapy	5	17	4	20	1	10
surgery	14	47	9	45	5	50

^a^CNS, central nervous system.

^b^others include Wilms tumor (2), soft tissue tumor (2), hepatoblastoma (1), fallopian tube carcinoma (1) osteosarcoma (1) Langerhans cell histiocytosis (1) teratoma (1).

We identified four domains in which parents use support for themselves and/or their families after the end of intensive therapy: (1) physical health, (2) emotional and mental health, (3) family functioning, and (4) practical support. Further, some barriers were mentioned.

### Physical health

All participants were taking their children for regular medical follow-up examinations. According to parents, some survivors received additional examinations such as hearing tests and hormone treatments when indicated. Almost every survivor participated in a FOR and received physiotherapy after primary therapy to promote physical recovery. Sometimes, hippo therapy supplemented physiotherapy. Survivors used assistive devices such as orthotics, hearing aids, or nursing support when needed. In most cases, Health Care Professionals (HCPs) actively recommended medical follow-up treatments.

Regardless of location, parents indicated a high level of satisfaction with the HCPs and the effect of the therapies. Nearly all respondents expressed high level of trust in the expertise of HCPs. The majority felt, that their needs were understood and that they received adequate medical support.

*“We were, on the whole, satisfied. When we had questions, we could ask, and sometimes we just accepted things. Because, after all, they couldn’t be changed anyway. You also have to have a bit of trust that they know what they’re doing. So, in that sense, we always had trust.” (mother of an 11-year-old boy)*

Follow-up appointments were often highly psychologically distressing due to the uncertainty surrounding remission. Frequent changes in HCPs during follow-ups were perceived as disruptive, with parents preferring a consistent practitioner for their child’s needs to feel safer and better understood. Some felt overwhelmed with managing their children’s medical appointments, long waiting periods, and understanding of examination results.

*“That perhaps in addition to medical guidance, there would be a contact person who looks at the child’s history a bit more comprehensively. So that you don’t feel so much like you’re solely responsible for keeping track of all possible consequences. Someone professional.” (mother of a 6-year-old girl)*

Parents occasionally expressed concerns about the side effects of the required control examinations, e.g., contrast agent in imaging techniques. Here, they wished for more comprehensive and individualized information. Multiple times, the desire for centralized and personalized care was expressed.

### Emotional and mental health

Although the majority of respondents reported psychological distress, only a small proportion sought psychosocial support. Mostly, utilization was limited to interventions offered during a FOR. Nonetheless, some participants had received regular psychological/psychotherapeutic treatment to receive support in coping with the disease or dealing with one’s own fears and burden. A few attended group offers for relatives of cancer survivors. In the majority of cases, parents took the initiative for psychological support themselves. Survivors and siblings mainly received occupational therapy followed by occasional support groups. To cope with the challenges of their illness, to receive emotional support, and to encourage social interaction, some adolescent survivors, and occasionally their siblings, joined youth camps with fellow survivors and their siblings. Those were organized and recommended by various entities such as the treating clinics, FORs, parent associations, or other providers.

*“I don’t know how to help him, so I think that’s up to the hospital. For example, my son participated in a sailing camp organized by the oncology department, which I believe was an excellent form of follow-up service. He was able to interact with other ill children on a completely different level, it was really beneficial for him.” (father of a 15-year-old boy)*

Some families make use of reintegration aids for school or kindergarten with the intention of strengthening the children’s social skills. These include, for example, home education, preparatory meetings with teachers, and school assistance. Together with occupational therapy, this was used to help overcome the fear of injections, touch, strangers, and loud noises. The siblings’ ongoing concern and fear of losing the survivor prompted the parents to desire additional professional support in coping with illness and emotional challenges.

Despite the predominantly positive feedback and high satisfaction among parents with the available services, there were nonetheless some parents who found certain aspects of psychosocial care to be insufficient. This was partly due to limited availability and partly because they felt that their family’s needs were not fully addressed. Some individuals expressed interest in a second inpatient rehabilitation after a period to address any newly surfaced mental or physical concerns. Parents in our study often reported lacking contact with other affected families near their home and wished for more exchange.

### Family functioning

Families benefited from family-centered rehabilitation measures. They expressed the importance of interaction with other families with cancer survivors. Parents sometimes experience tension within family members. For example, due to unequal processing of illness, less time together and unequal distribution of roles, partnership conflicts occurred more frequently. To resolve conflicts, some sought couple’s therapy. Psychotherapeutic or occupational therapy services for siblings were utilized to overcome conflicts arising from jealousy and unequal distribution of attention. While the majority of respondents reported being satisfied with the perceived care offers, some wished for more proactivity of health care providers and better involvement of siblings in psychosocial follow-up care.

*“I would just keep the care for my son as it is now; it’s perfect and working well. For my daughter, I would like her to receive emotional support and learn how to cope with her feelings. For the parents, I would wish for more support because I believe many couples handle it differently. When you’re faced with extreme situations in your daily life, you discover a completely different side of your partner. I think that couples therapy can help in this regard, ensuring that the family stays together after everything. Nothing would be worse than the child becoming healthy again, but the family falling apart.” (mother of a 9-year-old survivor and an 11-year-old sibling)*

### Practical support

Parents expressed a high need for practical support during follow-up care. Some were overwhelmed with forms and applications, e.g., regarding care support. In addition, a high number of follow-up appointments and therefore a high level of personal responsibility were reported to be burdensome. They particularly wished for improved appointment coordination and centralization of services to reduce the multitude of appointments at different locations and long waiting times.

*“Yes, time for yourself, but that’s hard to demand, I’d say. So go swimming in the evening, but you’re so tired yourself, you can’t do it anymore, because the day itself is so exhausting.” (mother of a 9-year-old girl)*

Parents have an intense demand for information about the further course of treatment as well as long-term effects and follow-up services. Specifically, services and psychosocial associations were found to help support parents with administrative tasks and applications. Participants who utilized psychosocial services reported feeling well supported and informed by the professionals. Psychosocial services were also consulted in terms of nutrition. These were particularly helpful in coordinating necessary applications, communicating with the health insurance company, and getting information on support services. However, parents desired more support at home and/or with childcare. In a few cases, families have received offers in the form of childcare or cleaning assistance. Few interviewees reported financial difficulties and an increased need for financial support due to childcare and increased treatment costs.

### Barriers

Parents mentioned their lack of awareness about available services as a significant barrier, limiting their access to necessary support. Although psychosocial services are usually provided during primary therapy, parents need to take initiative to get access to psychosocial support during follow-up care. However, when HCPs actively recommended services, families were more inclined to utilize them. Bureaucratic hurdles and lengthy waiting times had caused some parents to give up on accessing these services.

*“But the hurdles are also great, you are so caught up in your everyday life that you simply don’t know whom to turn to, I know that these procedures take a very long time and involve an incredible amount of administrative work, so you somehow don’t have the leisure to tackle it.” (father of a 6-year-old-girl)*

Another significant factor was lack of time. Especially families who live in remote areas experienced difficulties accessing facilities that offer psychosocial support services. Those families often reported a lack of specialized services that meet the specific needs of cancer survivors. Occasionally, caregivers claimed financial restrictions, especially for services that were not covered by health insurance. Locally unavailable or insufficient childcare care services was another important factor. Some survivors refused to receive psychosocial support, even if parents considered it as necessary. One reason was that they wanted to come to terms with the disease as soon as possible after primary therapy and therefore refused further offers.

## Discussion

Our findings indicate that physical needs of survivors are generally well addressed during follow-ups. Children received physical therapy and nutritional counseling as well as hearing and vision tests when needed. Wu et al. found that such services have a positive influence on further development.^24^ This is also reflected in the results of our study. Parents predominantly reported a high level of satisfaction with the medical follow-up care and the effectiveness of offers that address the long-term physical needs of their children. However, our interviews underline the explicit desire for a permanent contact person due to the uncertainties that arise when treatment providers change frequently. Continuity in HCPs benefits caregivers of children with chronic or life-threatening conditions, enhancing both satisfaction and disease understanding.^25^

An important observation is the persistence of unmet psychosocial needs, especially in the domains of information and family-centered care. Our study aligns with previous research that has identified unmet information needs as a critical concern among childhood cancer survivors. Unfulfilled information needs have been associated with a decreased quality of life for survivors and their families in the long term.^26^

Another relevant finding is the prevalence of family conflicts after primary therapy and a high need for partnership support due to exhaustion, different coping strategies and perceived neglect of siblings. Family cohesion, including parent-child and sibling relationships is crucial in the recovery process and the prevention of psychological long-term consequences for survivors and their families.^27^ Hence, guaranteeing access to family-orientated support (e.g., couple’s therapy, support groups for siblings) is important for the preservation of the family functionality and thus the mental health of all family members. Various studies describe promising results of family-oriented interventions.^1^ The present study has shown that support availed of was generally perceived as helpful and satisfactory. However, some families do not seek psychosocial support, even when experiencing limiting burdens, especially after primary treatment.

The initial focus on and prioritization of medical follow-up care might be caused by parental fears of progression and permanent physical limitations of their children. In addition to daily tasks and regular medical check-ups, caregivers reported a lack of resources to gather information about available services. A high degree of self-initiative is necessary after and during the transition to follow-up care. HCPs may not always effectively communicate information about available psychosocial services, leading to underutilization. Hence, caregivers are not aware of available psychosocial support services, or there may be limited access to these resources due to geographical or healthcare system constraints.

Furthermore, Psychosocial needs are multifaceted and can vary widely among survivors and their families.^7^ Tailoring support to meet these diverse needs can be challenging. One possible approach to improving follow-up care is the establishment of a centralized and interdisciplinary follow-up care. Initial reports from a Swiss study on unmet needs among childhood cancer survivors demonstrate that appointment coordination, communication among healthcare providers, as well as personalization of treatment, are enhanced.^18^

### Limitations

When interpreting the results, it is important to consider the following limitations. The findings of this study rely on individual reports. Some participants were recruited via psychosocial follow-up programs, which may lead to selection bias. In addition, a large proportion of the respondents were located in northern Germany. Since psychosocial follow-up care is structured differently internationally, the results can only be generalized to a limited extent. Only participants with sufficient knowledge of German were interviewed. Lastly, it is important to note that there were overlaps of the study with the corona pandemic and corresponding limitations.

## Conclusion

Families of childhood cancer survivors require a wide range of medical and psychosocial support services to cope with the challenges associated with the reintegration into everyday life. Despite available psychosocial services, limited utilization highlights the need to investigate and address access barriers. A holistic approach that includes the whole family can improve psychosocial support and contribute to strengthening the family union. Although parents are generally satisfied with medical and psychosocial services, they desire less rotation of HCPs, active provision of services, and personalized content to their specific needs. It is important to design medical and psychosocial support services in a needs-based, accessible, and flexible manner to ensure that they meet the needs of families and that families have the opportunity to access support when they need it. The outcomes of this study have the potential to empower healthcare providers in enhancing their ability to align their support with the unique requirements of families and streamline the accessibility of services.

### Clinical implications

Several clinical implications can be inferred from the barriers that families of children with cancer face in accessing psychosocial support services. Firstly, psychosocial support services should be better tailored to the needs and preferences of families, including accommodating their life circumstances and schedules. Flexibility and accessibility are key factors in ensuring that families receive the support they need. Secondly, psychosocial and medical support services should be actively addressed. This includes information and education campaigns to inform families about available resources and alleviate their concerns and reservations. Peer support, from individuals who have had similar experiences, can also be helpful. Thirdly, support services should be centralized and family-orientated. It is important that all family members (childhood cancer survivor, siblings, and parents) receive the support in coping with the disease and its consequences.

## Supplementary information

Supplementary information

## Data Availability

Anonymised transcripts that support the findings of this study are available from the corresponding author upon reasonable request.
